# Bis(1,10-phenanthroline-κ^2^ *N*,*N*′)(sulfato-*O*)copper(II) propane-1,3-diol monosolvate

**DOI:** 10.1107/S1600536812047721

**Published:** 2012-11-28

**Authors:** Kai-Long Zhong

**Affiliations:** a Department of Applied Chemistry, Nanjing College of Chemical Technology, Nanjing 210048, People’s Republic of China

## Abstract

In the title compound, [Cu(SO_4_)(C_12_H_8_N_2_)_2_]·C_3_H_8_O_2_, the Cu^II^ ion is bonded to two chelating 1,10-phenanthroline (phen) ligands and one O atom from a monodentate sulfate ligand in a distorted square-based pyramidal arrangement, with the O atom in a basal site. The two chelating N_2_C_2_ groups subtend a dihedral angle of 71.10 (15)°. In the crystal, the solvent mol­ecule forms two O—H⋯O hydrogen bonds to its adjacent complex mol­ecule. The chosen crystal was found to be a racemic twin; the presence of pseudosymmetry in the structure suggests the higher symmetry space group *C*2/*c*, but attempts to refine the structure in this space group resulted in an unsatisfactory model and high *R* and *wR* values.

## Related literature

For the ethane-1,2-diol solvate of the title complex, see: Zhong (2011*a* ▶) and for the propane-1,2-diol solvate of the title complex, see: Zhong (2011*b* ▶). For related structures of five-coordinate copper complexes and background references, see: Murphy & Hathaway (2003 ▶); Potočňák *et al.* (2008 ▶).
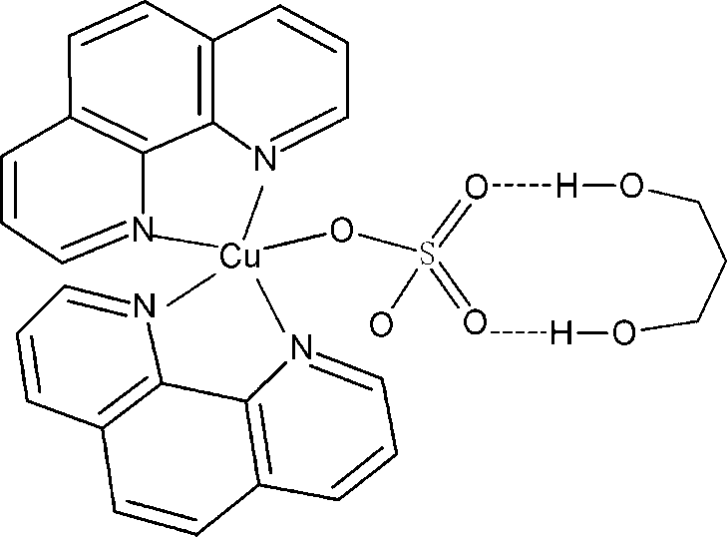


## Experimental

### 

#### Crystal data

[Cu(SO_4_)(C_12_H_8_N_2_)_2_]·C_3_H_8_O_2_
*M* *_r_* = 596.10
Monoclinic, 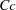

*a* = 17.523 (4) Å
*b* = 12.562 (3) Å
*c* = 13.438 (3) Å
β = 123.44 (3)°
*V* = 2468.4 (13) Å^3^
*Z* = 4
Mo *K*α radiation
μ = 1.02 mm^−1^
*T* = 223 K
0.30 × 0.20 × 0.20 mm

#### Data collection

Rigaku Mercury CCD diffractometer
Absorption correction: multi-scan (*REQAB*; Jacobson, 1998 ▶) *T*_min_ = 0.750, *T*_max_ = 1.000
6895 measured reflections
4049 independent reflections
3892 reflections with *I* > 2σ(*I*)
*R*_int_ = 0.017

#### Refinement

*R*[*F* ^2^ > 2σ(*F* ^2^)] = 0.036
*wR*(*F* ^2^) = 0.094
*S* = 1.07
4049 reflections
354 parameters
2 restraints
H-atom parameters constrained
Δρ_max_ = 0.70 e Å^−3^
Δρ_min_ = −0.82 e Å^−3^
Absolute structure: Flack (1983 ▶), 1224 Friedel pairs
Flack parameter: 0.56 (1)

### 

Data collection: *CrystalClear* (Rigaku, 2007 ▶); cell refinement: *CrystalClear*; data reduction: *CrystalClear*; program(s) used to solve structure: *SHELXS97* (Sheldrick, 2008 ▶); program(s) used to refine structure: *SHELXL97* (Sheldrick, 2008 ▶); molecular graphics: *XP* in *SHELXTL* (Sheldrick, 2008 ▶); software used to prepare material for publication: *SHELXTL*.

## Supplementary Material

Crystal structure: contains datablock(s) global, I. DOI: 10.1107/S1600536812047721/hb6990sup1.cif

Structure factors: contains datablock(s) I. DOI: 10.1107/S1600536812047721/hb6990Isup2.hkl

Additional supplementary materials:  crystallographic information; 3D view; checkCIF report

## Figures and Tables

**Table 1:** Selected bond lengths (Å)

Cu1—O1	1.956 (3)
Cu1—N1	2.001 (3)
Cu1—N3	2.009 (3)
Cu1—N2	2.071 (3)
Cu1—N4	2.175 (4)

**Table 2:** Hydrogen-bond geometry (Å, °)

*D*—H⋯*A*	*D*—H	H⋯*A*	*D*⋯*A*	*D*—H⋯*A*
O5—H5*B*⋯O3	0.82	1.99	2.788 (4)	166
O6—H6*B*⋯O4	0.82	2.01	2.817 (5)	166

**Fig. 1.**
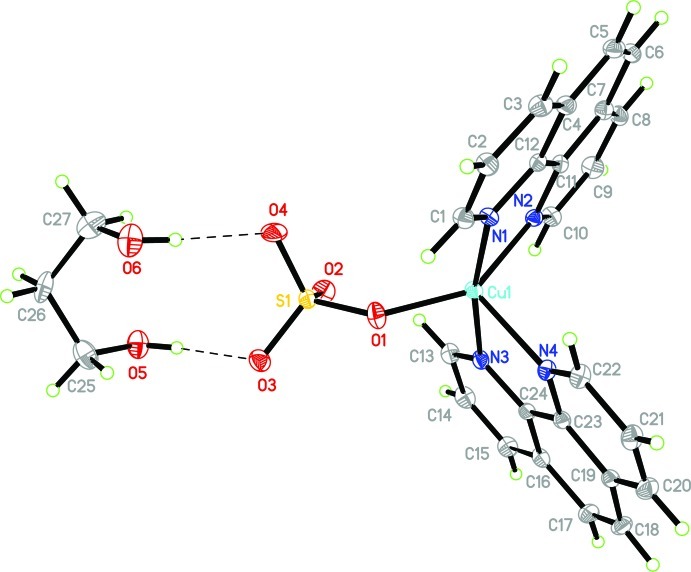
The molecular structure showing displacement ellipsoids drawn at the 35% probability level. Hydrogen bonds O—H···O are shown as dashed lines.

## supplementary crystallographic information

### Comment

Understanding the shape of coordination polyhedral in the case of five-coordination in the coordination chemistry has been caused much attention in the past few years (Murphy & Hathaway, 2003; Potočňák *et al.*, 2008). The title compound, (I), was unexpectedly obtained *via* a alcohol-solvothermal reaction and its crystal structure is now described.

The title complex is isostructural to the previously reported [CuSO_4_(C_12_H_8_N_2_)_2_].C_2_H_6_O_2_, (II), (Zhong, 2011*a*) and Cu-Phen complex with propane-1,2-diol monosolvate, (III), (Zhong, 2011*b*). In the title compound, X-ray diffraction experiment revealed that the Cu^II^ metal ion is five-coordinated in a distorted square-pyramidal manner by four N atoms(N1, N2, N3 and N4) from two chelating phen ligands and an O atoms(O1) from a monodentate sulfate ligand, the N1, N2, N3 and N4 atoms comprise a square, and the O1 atom site the apex of a square pyramid surrounding each metal atom. The Cu—O bond distance [1.956 (3) Å], the Cu—N bond distance [2.001 (3) - 2.175 (4) Å], and the N—Cu—N bite angle [80.09 (14) - 81.16 (14)°] are in good agreement with that observed in (II) and (III) (Table 1). The two chelating N2C2 groups are oriented at 71.10 (15)°, this is almost equal to that reported in (II) [71.1 (2)°] and smaller than that found in (III) [84.9 (4)°]. In the crystal, the neutral monomeric complex [CuSO_4_(C_12_H_8_N_2_)_2_] and solvent propane-1,3-diol components of (I) are connected by a pair of intermolecular O—H···O hydrogen bonding with the uncoordinated O atoms of the sulfate group(Table 2 & Fig. 1).

### Experimental

0.2 mmol phen, 0.1 mmol melamine, 0.1 mmol CuSO_4_.5H_2_O, 2.0 ml propane-1,3-diol and 1.0 ml water were mixed and placed in a thick Pyrex tube, which was sealed and heated to 453 K for 96 h, whereupon blue block-shaped crystals of (I) were obtained.

### Refinement

The presence of pseudo-symmetry in the structure suggests a higher symmetry space group *C*2/*c*. But attempts to refine the structure in the space group *C*2/*c* resulted in an unsatisfactory model and high *R* and *wR* values. Hence the requirement to solve in *Cc*. The reported Flack parameter was refined as s full least-squares and obtained by TWIN/BASF procedure in *SHELXL* (Sheldrick, 2008).

The H atoms of phen were positioned geometrically and allowed to ride on their parent atoms, with C—H = 0.93 Å and *U*_iso_(H) = 1.2*U*_eq_(C). The H atoms of propane-1,3-diol were placed in geometrically idealized positions and refined as riding atoms, with C—H = 0.97 Å and O—H = 0.82 Å; *U*_iso_(H) = 1.2*U*_eq_(C) and 1.5*U*_eq_(O).

### Crystal data

**Table:**

[Cu(SO_4_)(C_12_H_8_N_2_)_2_]·C_3_H_8_O_2_	*F*(000) = 1228
*M**_r_* = 596.10	*D*_x_ = 1.604 Mg m^−^^3^
Monoclinic, *C**c*	Mo *K*α radiation, λ = 0.71073 Å
Hall symbol: C -2yc	Cell parameters from 6081 reflections
*a* = 17.523 (4) Å	θ = 3.1–27.5°
*b* = 12.562 (3) Å	µ = 1.02 mm^−^^1^
*c* = 13.438 (3) Å	*T* = 223 K
β = 123.44 (3)°	Block, blue
*V* = 2468.4 (13) Å^3^	0.30 × 0.20 × 0.20 mm
*Z* = 4	

### Data collection

**Table:**

Rigaku Mercury CCD diffractometer	4049 independent reflections
Radiation source: fine-focus sealed tube	3892 reflections with *I* > 2σ(*I*)
Graphite monochromator	*R*_int_ = 0.017
Detector resolution: 28.5714 pixels mm^-1^	θ_max_ = 27.5°, θ_min_ = 3.1°
ω scan	*h* = −22→20
Absorption correction: multi-scan (REQAB; Jacobson, 1998)	*k* = −15→15
*T*_min_ = 0.750, *T*_max_ = 1.000	*l* = −17→15
6895 measured reflections	

### Refinement

**Table:**

Refinement on *F*^2^	Hydrogen site location: inferred from neighbouring sites
Least-squares matrix: full	H-atom parameters constrained
*R*[*F*^2^ > 2σ(*F*^2^)] = 0.036	*w* = 1/[σ^2^(*F*_o_^2^) + (0.0678*P*)^2^ + 0.9364*P*] where *P* = (*F*_o_^2^ + 2*F*_c_^2^)/3
*wR*(*F*^2^) = 0.094	(Δ/σ)_max_ = 0.002
*S* = 1.07	Δρ_max_ = 0.70 e Å^−^^3^
4049 reflections	Δρ_min_ = −0.82 e Å^−^^3^
354 parameters	Extinction correction: *SHELXL97* (Sheldrick, 2008), Fc^*^=kFc[1+0.001xFc^2^λ^3^/sin(2θ)]^-1/4^
2 restraints	Extinction coefficient: 0.0074 (6)
Primary atom site location: structure-invariant direct methods	Absolute structure: Flack (1983), 1224 Friedel pairs
Secondary atom site location: difference Fourier map	Flack parameter: 0.56 (1)

### Special details

**Table:**

Geometry. All e.s.d.'s (except the e.s.d. in the dihedral angle between two l.s. planes) are estimated using the full covariance matrix. The cell e.s.d.'s are taken into account individually in the estimation of e.s.d.'s in distances, angles and torsion angles; correlations between e.s.d.'s in cell parameters are only used when they are defined by crystal symmetry. An approximate (isotropic) treatment of cell e.s.d.'s is used for estimating e.s.d.'s involving l.s. planes.
Refinement. Refinement of *F*^2^ against ALL reflections. The weighted *R*-factor *wR* and goodness of fit *S* are based on *F*^2^, conventional *R*-factors *R* are based on *F*, with *F* set to zero for negative *F*^2^. The threshold expression of *F*^2^ > σ(*F*^2^) is used only for calculating *R*-factors(gt) *etc*. and is not relevant to the choice of reflections for refinement. *R*-factors based on *F*^2^ are statistically about twice as large as those based on *F*, and *R*- factors based on ALL data will be even larger.

### Fractional atomic coordinates and isotropic or equivalent isotropic displacement parameters (Å^2^)

**Table:**

	*x*	*y*	*z*	*U*_iso_*/*U*_eq_	
Cu1	0.27326 (3)	0.20114 (3)	0.12409 (3)	0.01905 (12)	
S1	0.26222 (7)	−0.04682 (6)	0.13203 (9)	0.0231 (2)	
O1	0.24083 (19)	0.0554 (2)	0.0634 (2)	0.0302 (6)	
O2	0.27343 (19)	−0.0240 (2)	0.2470 (2)	0.0320 (6)	
O3	0.1834 (2)	−0.1175 (2)	0.0615 (3)	0.0289 (6)	
O4	0.3462 (2)	−0.0955 (3)	0.1531 (3)	0.0384 (8)	
O5	0.2035 (3)	−0.3097 (2)	0.1774 (3)	0.0356 (8)	
H5B	0.1993	−0.2483	0.1543	0.053*	
O6	0.3321 (3)	−0.2976 (2)	0.0534 (3)	0.0402 (9)	
H6B	0.3338	−0.2348	0.0713	0.060*	
N1	0.3609 (2)	0.2146 (2)	0.0734 (3)	0.0182 (7)	
N2	0.3697 (2)	0.2982 (2)	0.2604 (3)	0.0187 (7)	
N3	0.1833 (2)	0.2196 (2)	0.1711 (3)	0.0211 (7)	
N4	0.1724 (3)	0.3012 (2)	−0.0218 (3)	0.0200 (7)	
C1	0.3546 (3)	0.1723 (3)	−0.0219 (3)	0.0231 (8)	
H1A	0.3088	0.1225	−0.0664	0.028*	
C2	0.4122 (3)	0.1985 (3)	−0.0580 (4)	0.0238 (9)	
H2A	0.4048	0.1674	−0.1256	0.029*	
C3	0.4815 (3)	0.2722 (3)	0.0085 (4)	0.0246 (8)	
H3A	0.5207	0.2913	−0.0148	0.030*	
C4	0.4922 (3)	0.3180 (3)	0.1117 (4)	0.0220 (8)	
C5	0.5640 (3)	0.3893 (3)	0.1891 (4)	0.0246 (8)	
H5A	0.6067	0.4091	0.1719	0.030*	
C6	0.5707 (3)	0.4287 (3)	0.2877 (4)	0.0242 (9)	
H6A	0.6178	0.4755	0.3372	0.029*	
C7	0.5067 (3)	0.3993 (3)	0.3165 (3)	0.0213 (7)	
C8	0.5099 (3)	0.4370 (3)	0.4191 (4)	0.0239 (8)	
H8A	0.5559	0.4833	0.4722	0.029*	
C9	0.4455 (3)	0.4046 (4)	0.4383 (4)	0.0292 (9)	
H9A	0.4473	0.4287	0.5050	0.035*	
C10	0.3765 (3)	0.3353 (3)	0.3588 (3)	0.0232 (8)	
H10A	0.3333	0.3138	0.3745	0.028*	
C11	0.4352 (3)	0.3301 (3)	0.2419 (3)	0.0180 (7)	
C12	0.4292 (3)	0.2867 (3)	0.1391 (4)	0.0172 (8)	
C13	0.1905 (3)	0.1780 (4)	0.2671 (4)	0.0253 (8)	
H13A	0.2369	0.1293	0.3136	0.030*	
C14	0.1285 (4)	0.2067 (3)	0.2998 (5)	0.0282 (10)	
H14A	0.1355	0.1784	0.3684	0.034*	
C15	0.0597 (3)	0.2749 (3)	0.2311 (4)	0.0265 (9)	
H15A	0.0187	0.2932	0.2517	0.032*	
C16	0.0500 (3)	0.3184 (3)	0.1289 (4)	0.0221 (8)	
C17	−0.0231 (3)	0.3899 (3)	0.0491 (4)	0.0273 (9)	
H17A	−0.0653	0.4110	0.0664	0.033*	
C18	−0.0307 (3)	0.4266 (3)	−0.0513 (4)	0.0275 (9)	
H18A	−0.0796	0.4705	−0.1030	0.033*	
C19	0.0350 (3)	0.3991 (3)	−0.0794 (4)	0.0217 (7)	
C20	0.0297 (3)	0.4362 (3)	−0.1815 (4)	0.0295 (9)	
H20A	−0.0173	0.4816	−0.2346	0.035*	
C21	0.0948 (3)	0.4043 (3)	−0.2017 (4)	0.0274 (9)	
H21A	0.0918	0.4270	−0.2697	0.033*	
C22	0.1665 (3)	0.3369 (3)	−0.1190 (4)	0.0250 (8)	
H22A	0.2110	0.3169	−0.1328	0.030*	
C23	0.1081 (3)	0.3307 (3)	−0.0015 (3)	0.0194 (7)	
C24	0.1152 (3)	0.2895 (3)	0.1030 (4)	0.0197 (8)	
C25	0.1907 (4)	−0.3797 (4)	0.0891 (5)	0.0443 (12)	
H25A	0.1684	−0.3402	0.0160	0.053*	
H25B	0.1443	−0.4314	0.0739	0.053*	
C26	0.2772 (4)	−0.4380 (3)	0.1223 (6)	0.0428 (10)	
H26A	0.2953	−0.4846	0.1894	0.051*	
H26B	0.2648	−0.4822	0.0557	0.051*	
C27	0.3551 (4)	−0.3643 (4)	0.1545 (5)	0.0478 (13)	
H27A	0.3683	−0.3199	0.2212	0.057*	
H27B	0.4095	−0.4055	0.1784	0.057*	

### Atomic displacement parameters (Å^2^)

**Table:**

	*U*^11^	*U*^22^	*U*^33^	*U*^12^	*U*^13^	*U*^23^
Cu1	0.01945 (19)	0.01898 (19)	0.02378 (19)	−0.00159 (19)	0.01512 (15)	−0.0013 (2)
S1	0.0213 (5)	0.0179 (3)	0.0240 (4)	−0.0008 (4)	0.0087 (3)	−0.0011 (4)
O1	0.0415 (16)	0.0209 (12)	0.0289 (13)	−0.0051 (11)	0.0199 (12)	0.0000 (10)
O2	0.0374 (15)	0.0364 (15)	0.0205 (12)	0.0032 (12)	0.0149 (12)	0.0005 (11)
O3	0.0291 (15)	0.0270 (14)	0.0299 (14)	−0.0041 (11)	0.0158 (13)	0.0025 (11)
O4	0.0218 (15)	0.0423 (16)	0.056 (2)	−0.0006 (12)	0.0247 (15)	−0.0109 (15)
O5	0.051 (2)	0.0288 (15)	0.0388 (18)	−0.0008 (13)	0.0325 (17)	0.0025 (13)
O6	0.062 (2)	0.0354 (19)	0.043 (2)	0.0029 (14)	0.0413 (19)	0.0006 (13)
N1	0.0179 (17)	0.0201 (16)	0.0172 (16)	−0.0027 (12)	0.0100 (15)	−0.0017 (12)
N2	0.0177 (17)	0.0196 (17)	0.0179 (16)	0.0009 (11)	0.0093 (15)	0.0022 (11)
N3	0.0235 (19)	0.0175 (15)	0.0267 (19)	−0.0016 (13)	0.0167 (17)	−0.0002 (13)
N4	0.0241 (19)	0.0194 (18)	0.0202 (17)	0.0009 (12)	0.0145 (16)	0.0022 (12)
C1	0.023 (2)	0.0238 (18)	0.0231 (19)	−0.0027 (17)	0.0132 (17)	−0.0066 (17)
C2	0.026 (2)	0.024 (2)	0.026 (2)	−0.0010 (14)	0.017 (2)	−0.0043 (15)
C3	0.025 (2)	0.031 (2)	0.024 (2)	0.0026 (17)	0.0172 (18)	0.0021 (17)
C4	0.0194 (19)	0.0232 (18)	0.0225 (19)	0.0006 (16)	0.0110 (17)	0.0036 (16)
C5	0.020 (2)	0.028 (2)	0.0269 (19)	0.0011 (15)	0.0138 (17)	0.0037 (17)
C6	0.021 (2)	0.022 (2)	0.0263 (19)	−0.0022 (14)	0.0110 (17)	0.0021 (15)
C7	0.0219 (19)	0.0216 (18)	0.0185 (17)	0.0003 (15)	0.0099 (16)	0.0002 (15)
C8	0.025 (2)	0.021 (2)	0.0211 (17)	−0.0031 (15)	0.0095 (17)	−0.0036 (15)
C9	0.033 (2)	0.033 (2)	0.0174 (18)	−0.0021 (18)	0.0112 (19)	−0.0055 (16)
C10	0.027 (2)	0.026 (2)	0.0230 (19)	−0.0014 (16)	0.0177 (18)	−0.0017 (16)
C11	0.0199 (18)	0.0166 (16)	0.0190 (17)	0.0003 (14)	0.0116 (16)	0.0001 (15)
C12	0.0178 (19)	0.0170 (18)	0.0169 (18)	0.0017 (13)	0.0096 (16)	0.0014 (13)
C13	0.030 (2)	0.0236 (18)	0.029 (2)	−0.0012 (17)	0.021 (2)	−0.0020 (18)
C14	0.035 (3)	0.032 (2)	0.029 (2)	−0.0057 (16)	0.025 (2)	−0.0029 (15)
C15	0.029 (2)	0.028 (2)	0.033 (2)	−0.0009 (17)	0.024 (2)	−0.0052 (18)
C16	0.025 (2)	0.0209 (17)	0.028 (2)	−0.0048 (16)	0.0192 (18)	−0.0066 (17)
C17	0.022 (2)	0.026 (2)	0.037 (2)	0.0046 (16)	0.0182 (19)	−0.0045 (18)
C18	0.021 (2)	0.025 (2)	0.027 (2)	0.0065 (14)	0.0081 (18)	−0.0025 (16)
C19	0.0194 (19)	0.0185 (18)	0.0235 (18)	−0.0019 (15)	0.0094 (16)	−0.0047 (15)
C20	0.027 (2)	0.028 (2)	0.023 (2)	0.0028 (16)	0.0075 (18)	0.0029 (17)
C21	0.034 (2)	0.026 (2)	0.0211 (19)	0.0008 (17)	0.0148 (19)	0.0039 (16)
C22	0.031 (2)	0.024 (2)	0.0231 (19)	0.0028 (17)	0.0175 (18)	0.0023 (16)
C23	0.0188 (18)	0.0181 (17)	0.0190 (17)	−0.0019 (15)	0.0090 (16)	−0.0049 (15)
C24	0.018 (2)	0.0178 (18)	0.024 (2)	−0.0015 (13)	0.0115 (18)	−0.0029 (14)
C25	0.052 (3)	0.037 (3)	0.049 (3)	−0.014 (2)	0.031 (3)	−0.002 (2)
C26	0.059 (3)	0.0205 (15)	0.053 (2)	0.014 (2)	0.034 (2)	0.009 (3)
C27	0.043 (3)	0.052 (3)	0.046 (3)	0.011 (2)	0.022 (3)	0.006 (2)

### Geometric parameters (Å, º)

**Table:**

Cu1—O1	1.956 (3)	C8—C9	1.350 (6)
Cu1—N1	2.001 (3)	C8—H8A	0.9300
Cu1—N3	2.009 (3)	C9—C10	1.392 (6)
Cu1—N2	2.071 (3)	C9—H9A	0.9300
Cu1—N4	2.175 (4)	C10—H10A	0.9300
S1—O3	1.466 (3)	C11—C12	1.433 (6)
S1—O4	1.469 (3)	C13—C14	1.425 (6)
S1—O2	1.475 (3)	C13—H13A	0.9300
S1—O1	1.503 (3)	C14—C15	1.347 (7)
O5—C25	1.392 (6)	C14—H14A	0.9300
O5—H5B	0.8200	C15—C16	1.399 (6)
O6—C27	1.451 (6)	C15—H15A	0.9300
O6—H6B	0.8200	C16—C24	1.413 (6)
N1—C1	1.333 (5)	C16—C17	1.443 (6)
N1—C12	1.367 (5)	C17—C18	1.360 (6)
N2—C10	1.343 (5)	C17—H17A	0.9300
N2—C11	1.363 (5)	C18—C19	1.438 (6)
N3—C13	1.331 (6)	C18—H18A	0.9300
N3—C24	1.352 (6)	C19—C20	1.403 (6)
N4—C22	1.331 (5)	C19—C23	1.413 (6)
N4—C23	1.349 (5)	C20—C21	1.371 (6)
C1—C2	1.380 (6)	C20—H20A	0.9300
C1—H1A	0.9300	C21—C22	1.411 (6)
C2—C3	1.390 (6)	C21—H21A	0.9300
C2—H2A	0.9300	C22—H22A	0.9300
C3—C4	1.416 (6)	C23—C24	1.436 (6)
C3—H3A	0.9300	C25—C26	1.513 (7)
C4—C12	1.398 (6)	C25—H25A	0.9700
C4—C5	1.423 (6)	C25—H25B	0.9700
C5—C6	1.358 (6)	C26—C27	1.503 (8)
C5—H5A	0.9300	C26—H26A	0.9700
C6—C7	1.422 (6)	C26—H26B	0.9700
C6—H6A	0.9300	C27—H27A	0.9700
C7—C11	1.395 (6)	C27—H27B	0.9700
C7—C8	1.429 (5)		
			
O1—Cu1—N1	92.19 (12)	N2—C11—C7	124.4 (3)
O1—Cu1—N3	98.11 (12)	N2—C11—C12	116.3 (3)
N1—Cu1—N3	168.47 (9)	C7—C11—C12	119.3 (3)
O1—Cu1—N2	145.89 (12)	N1—C12—C4	123.6 (4)
N1—Cu1—N2	81.16 (14)	N1—C12—C11	116.6 (4)
N3—Cu1—N2	93.02 (14)	C4—C12—C11	119.8 (4)
O1—Cu1—N4	105.07 (12)	N3—C13—C14	121.2 (4)
N1—Cu1—N4	92.28 (13)	N3—C13—H13A	119.4
N3—Cu1—N4	80.09 (14)	C14—C13—H13A	119.4
N2—Cu1—N4	108.58 (9)	C15—C14—C13	119.7 (4)
O3—S1—O4	110.99 (16)	C15—C14—H14A	120.1
O3—S1—O2	109.25 (16)	C13—C14—H14A	120.1
O4—S1—O2	109.75 (19)	C14—C15—C16	120.0 (4)
O3—S1—O1	107.05 (16)	C14—C15—H15A	120.0
O4—S1—O1	111.03 (18)	C16—C15—H15A	120.0
O2—S1—O1	108.70 (15)	C15—C16—C24	117.7 (4)
S1—O1—Cu1	128.81 (16)	C15—C16—C17	123.4 (4)
C25—O5—H5B	109.5	C24—C16—C17	118.9 (4)
C27—O6—H6B	109.5	C18—C17—C16	120.7 (4)
C1—N1—C12	117.7 (4)	C18—C17—H17A	119.6
C1—N1—Cu1	128.3 (3)	C16—C17—H17A	119.6
C12—N1—Cu1	113.5 (3)	C17—C18—C19	121.5 (4)
C10—N2—C11	116.8 (3)	C17—C18—H18A	119.2
C10—N2—Cu1	131.4 (3)	C19—C18—H18A	119.2
C11—N2—Cu1	111.7 (3)	C20—C19—C23	118.1 (4)
C13—N3—C24	119.2 (4)	C20—C19—C18	123.1 (4)
C13—N3—Cu1	126.0 (3)	C23—C19—C18	118.8 (4)
C24—N3—Cu1	114.5 (3)	C21—C20—C19	118.8 (4)
C22—N4—C23	119.0 (4)	C21—C20—H20A	120.6
C22—N4—Cu1	131.6 (3)	C19—C20—H20A	120.6
C23—N4—Cu1	109.3 (3)	C20—C21—C22	119.8 (4)
N1—C1—C2	123.6 (4)	C20—C21—H21A	120.1
N1—C1—H1A	118.2	C22—C21—H21A	120.1
C2—C1—H1A	118.2	N4—C22—C21	121.9 (4)
C1—C2—C3	118.8 (4)	N4—C22—H22A	119.1
C1—C2—H2A	120.6	C21—C22—H22A	119.1
C3—C2—H2A	120.6	N4—C23—C19	122.2 (3)
C2—C3—C4	119.8 (4)	N4—C23—C24	117.8 (3)
C2—C3—H3A	120.1	C19—C23—C24	119.9 (3)
C4—C3—H3A	120.1	N3—C24—C16	122.1 (4)
C12—C4—C3	116.5 (4)	N3—C24—C23	117.7 (4)
C12—C4—C5	119.6 (4)	C16—C24—C23	120.1 (4)
C3—C4—C5	123.9 (4)	O5—C25—C26	113.0 (5)
C6—C5—C4	120.5 (4)	O5—C25—H25A	109.0
C6—C5—H5A	119.7	C26—C25—H25A	109.0
C4—C5—H5A	119.7	O5—C25—H25B	109.0
C5—C6—C7	121.0 (4)	C26—C25—H25B	109.0
C5—C6—H6A	119.5	H25A—C25—H25B	107.8
C7—C6—H6A	119.5	C27—C26—C25	112.9 (3)
C11—C7—C6	119.7 (3)	C27—C26—H26A	109.0
C11—C7—C8	116.3 (4)	C25—C26—H26A	109.0
C6—C7—C8	124.0 (4)	C27—C26—H26B	109.0
C9—C8—C7	119.4 (4)	C25—C26—H26B	109.0
C9—C8—H8A	120.3	H26A—C26—H26B	107.8
C7—C8—H8A	120.3	O6—C27—C26	110.3 (5)
C8—C9—C10	120.4 (4)	O6—C27—H27A	109.6
C8—C9—H9A	119.8	C26—C27—H27A	109.6
C10—C9—H9A	119.8	O6—C27—H27B	109.6
N2—C10—C9	122.7 (4)	C26—C27—H27B	109.6
N2—C10—H10A	118.7	H27A—C27—H27B	108.1
C9—C10—H10A	118.7		
			
O3—S1—O1—Cu1	144.4 (2)	C10—N2—C11—C12	177.6 (3)
O4—S1—O1—Cu1	−94.3 (2)	Cu1—N2—C11—C12	−3.8 (4)
O2—S1—O1—Cu1	26.5 (3)	C6—C7—C11—N2	−179.0 (3)
N1—Cu1—O1—S1	110.9 (2)	C8—C7—C11—N2	0.7 (6)
N3—Cu1—O1—S1	−74.3 (2)	C6—C7—C11—C12	2.1 (6)
N2—Cu1—O1—S1	33.5 (4)	C8—C7—C11—C12	−178.2 (4)
N4—Cu1—O1—S1	−156.1 (2)	C1—N1—C12—C4	−0.1 (6)
O1—Cu1—N1—C1	34.3 (3)	Cu1—N1—C12—C4	−172.7 (3)
N3—Cu1—N1—C1	−119.1 (8)	C1—N1—C12—C11	−179.3 (4)
N2—Cu1—N1—C1	−179.4 (4)	Cu1—N1—C12—C11	8.1 (4)
N4—Cu1—N1—C1	−70.9 (3)	C3—C4—C12—N1	1.2 (6)
O1—Cu1—N1—C12	−154.1 (3)	C5—C4—C12—N1	−176.6 (4)
N3—Cu1—N1—C12	52.5 (11)	C3—C4—C12—C11	−179.6 (4)
N2—Cu1—N1—C12	−7.7 (3)	C5—C4—C12—C11	2.6 (6)
N4—Cu1—N1—C12	100.7 (3)	N2—C11—C12—N1	−2.7 (5)
O1—Cu1—N2—C10	−94.7 (4)	C7—C11—C12—N1	176.2 (4)
N1—Cu1—N2—C10	−175.5 (4)	N2—C11—C12—C4	178.0 (4)
N3—Cu1—N2—C10	14.5 (4)	C7—C11—C12—C4	−3.0 (6)
N4—Cu1—N2—C10	95.1 (3)	C24—N3—C13—C14	−0.7 (6)
O1—Cu1—N2—C11	87.0 (3)	Cu1—N3—C13—C14	172.2 (3)
N1—Cu1—N2—C11	6.2 (2)	N3—C13—C14—C15	1.8 (6)
N3—Cu1—N2—C11	−163.8 (3)	C13—C14—C15—C16	−0.8 (6)
N4—Cu1—N2—C11	−83.2 (3)	C14—C15—C16—C24	−1.1 (6)
O1—Cu1—N3—C13	75.9 (3)	C14—C15—C16—C17	178.4 (4)
N1—Cu1—N3—C13	−131.1 (8)	C15—C16—C17—C18	−177.6 (4)
N2—Cu1—N3—C13	−71.8 (3)	C24—C16—C17—C18	1.8 (6)
N4—Cu1—N3—C13	179.8 (4)	C16—C17—C18—C19	−2.3 (6)
O1—Cu1—N3—C24	−110.9 (3)	C17—C18—C19—C20	−179.4 (4)
N1—Cu1—N3—C24	42.2 (11)	C17—C18—C19—C23	1.2 (6)
N2—Cu1—N3—C24	101.4 (3)	C23—C19—C20—C21	0.4 (6)
N4—Cu1—N3—C24	−6.9 (3)	C18—C19—C20—C21	−179.0 (4)
O1—Cu1—N4—C22	−80.9 (4)	C19—C20—C21—C22	−1.2 (7)
N1—Cu1—N4—C22	12.0 (4)	C23—N4—C22—C21	−0.3 (6)
N3—Cu1—N4—C22	−176.7 (4)	Cu1—N4—C22—C21	−178.4 (3)
N2—Cu1—N4—C22	93.4 (4)	C20—C21—C22—N4	1.2 (7)
O1—Cu1—N4—C23	100.9 (2)	C22—N4—C23—C19	−0.6 (6)
N1—Cu1—N4—C23	−166.2 (2)	Cu1—N4—C23—C19	177.9 (3)
N3—Cu1—N4—C23	5.1 (2)	C22—N4—C23—C24	178.9 (3)
N2—Cu1—N4—C23	−84.8 (3)	Cu1—N4—C23—C24	−2.6 (4)
C12—N1—C1—C2	−0.8 (6)	C20—C19—C23—N4	0.5 (6)
Cu1—N1—C1—C2	170.5 (3)	C18—C19—C23—N4	180.0 (3)
N1—C1—C2—C3	0.6 (6)	C20—C19—C23—C24	−179.0 (4)
C1—C2—C3—C4	0.6 (6)	C18—C19—C23—C24	0.5 (6)
C2—C3—C4—C12	−1.4 (6)	C13—N3—C24—C16	−1.3 (6)
C2—C3—C4—C5	176.3 (4)	Cu1—N3—C24—C16	−175.0 (3)
C12—C4—C5—C6	−1.3 (6)	C13—N3—C24—C23	−178.4 (4)
C3—C4—C5—C6	−178.9 (4)	Cu1—N3—C24—C23	7.8 (4)
C4—C5—C6—C7	0.4 (6)	C15—C16—C24—N3	2.2 (6)
C5—C6—C7—C11	−0.8 (6)	C17—C16—C24—N3	−177.3 (4)
C5—C6—C7—C8	179.5 (4)	C15—C16—C24—C23	179.3 (4)
C11—C7—C8—C9	0.0 (6)	C17—C16—C24—C23	−0.2 (6)
C6—C7—C8—C9	179.7 (4)	N4—C23—C24—N3	−3.2 (5)
C7—C8—C9—C10	−0.1 (6)	C19—C23—C24—N3	176.3 (4)
C11—N2—C10—C9	1.2 (6)	N4—C23—C24—C16	179.6 (4)
Cu1—N2—C10—C9	−177.1 (3)	C19—C23—C24—C16	−0.9 (6)
C8—C9—C10—N2	−0.5 (7)	O5—C25—C26—C27	−55.4 (6)
C10—N2—C11—C7	−1.3 (6)	C25—C26—C27—O6	−61.9 (6)
Cu1—N2—C11—C7	177.3 (3)		

### Hydrogen-bond geometry (Å, º)

**Table:**

*D*—H···*A*	*D*—H	H···*A*	*D*···*A*	*D*—H···*A*
O5—H5*B*···O3	0.82	1.99	2.788 (4)	166
O6—H6*B*···O4	0.82	2.01	2.817 (5)	166
